# Theoretical–Methodological Foundations for the Global Integration Method (Método de Integração Global—MIG) in the Treatment of Autism Spectrum Disorder

**DOI:** 10.3390/children11020191

**Published:** 2024-02-02

**Authors:** Renato Guimarães Loffi, Thalita Karla Flores Cruz, Giulia Moreira Paiva, Deisiane Oliveira Souto, Simone Rosa Barreto, Patrícia Aparecida Neves Santana, Amanda Aparecida Alves Cunha Nascimento, Fabiana Rachel Martins Costa, Elisa Braz Cota, Vitor Geraldi Haase

**Affiliations:** 1 Instituto de Neurodesenvolvimento, Cognição e Educação Inclusiva (INCEI), TREINITEC Ltda., Rua Carmélia Loffi 17, Justinópolis, Ribeirão das Neves 33900-730, MG, Brazil; renatoloffi@treinitec.com.br (R.G.L.); giuliapaiva@treinitec.com.br (G.M.P.); deisianesouto@treinitec.com.br (D.O.S.); simonebarreto@treinitec.com.br (S.R.B.); patricianeves@treinitec.com.br (P.A.N.S.); amandanascimento@treinitec.com.br (A.A.A.C.N.); fabianamartins@treinitec.com.br (F.R.M.C.); elisacota@treinitec.com.br (E.B.C.); vitorghaase@treinitec.com.br (V.G.H.); 2 Programa de Pós-Graduação em Neurociências, Universidade Federal de Minas Gerais, Belo Horizonte 31270-901, MG, Brazil; 3 Programa de Pós-Graduação em Ciências Fonoaudiológicas, Universidade Federal de Minas Gerais, Belo Horizonte 31270-901, MG, Brazil; 4 Programa de Pós-Graduação em Psicologia: Cognição e Comportamento, Universidade Federal de Minas Gerais, Belo Horizonte 31270-901, MG, Brazil

**Keywords:** autism spectrum disorder, intensive intervention, interdisciplinary program, predictive coding, embodied cognition, therapeutic suit

## Abstract

Currently, there is no intervention model for autism spectrum disorder (ASD) that addresses all levels and factors of the International Classification of Functioning, Disability and Health (ICF, WHO). The most researched programs focus on naturalistic, developmental and behavioral approaches to socio-communication. Less attention has been paid to motor and environmental reactivity aspects (behavior/interest restriction and sensory reactivity). The evidence rationale for the Global Integration Method (MIG, “Método de Integração Global”), a model addressing sensorimotor reactivity in addition to socio-communication, is presented. MIG is an integrative, interdisciplinary, family-oriented intervention and naturalistic program that addresses all levels and moderating factors of ASD’s impact. MIG’s theoretical rationale is based on the predictive coding impairment and embodied cognition hypotheses. MIG incorporates both bottom-up (flexible therapeutic suit, social-motor synchronization) and top-down (schematic social information processing, narratives, imagery) strategies to promote the building and use of accurate, flexible and context-sensitive internal predictive models. MIG is based on the premises that predictive coding improves both socio-communication and environmental reactivity, and that the postural stabilization provided by the flexible therapeutic suit frees information processing resources for socio-cognitive learning. MIG builds on interdisciplinary, professionally and parentally mediated work based on behavioral principles of intensive training in a situated environment.

## 1. Introduction

Autism spectrum disorder (ASD) is usually a lifelong neurodevelopmental disorder with presumed genetic, environmental and epigenetic causes that influences socialization and communication, in addition to exhibiting restricted, repetitive and stereotyped behavior patterns [1]. People with ASD may have limited involvement in social interactions with peers and restrictions in participation at home, school and in the community. Children and adolescents with ASD may present higher prevalence of intellectual disability, self-injurious behavior, sensory/perceptual difficulties and emotional problems compared to those with typical development [2]. The current literature also highlights the presence of motor abnormalities in ASD, with approximately 80% of children presenting manifestations in different aspects of the motor system [3,4]. Generally, ASD manifests before the age of 3 and results in lifelong challenges for an individual. ASD can manifest in individuals from different ethnic and socioeconomic groups and is more likely to occur in males than females [5]. ASD challenges families and professionals to find effective interventions that can improve functionality and quality of life for individuals throughout their lifespan.

Several interventions have been proposed to help people with ASD and their families [6,7]. In general, the main evidence-based interventions focus on socio-behavioral symptoms [2]. Most children with ASD undergo interventions based on the applied behavioral analysis approach—ABA [4]. Some examples include Naturalistic Developmental Behavioral Interventions—NDBI [8] and Treatment and Education of Autistic and Related Communication Disabilities Children—TEACCH [9]. Data from a large USA study involving 11.814 children with ASD (SPARK study) [10] showed that 67.8% of people with ASD received ABA-based behavioral therapies and 47.7% received social skills interventions. Only 32% of children in the SPARK study received physical therapy for motor dysfunction. An intervention model that strictly emphasizes behavioral or social changes may limit access to interventions necessary to comprehensively meet the needs of children and adolescents with ASD. Therefore, integrated, interdisciplinary and intensive interventions are still needed to address all domains relevant for this population.

The present article aims to introduce and explain the theoretical–methodological bases that guide the Global Integration Method (Método de Integração Global, MIG), an interdisciplinary and intensive intervention program for autism spectrum disorder (ASD, or autism hereinafter). MIG was developed at TREINITEC Ltda. (Ribeirão das Neves, Brazil) based on the work of physical therapist Renato Loffi and his team. The MIG framework was developed mainly considering the needs of children and adolescents requiring levels 2 and 3 of support [1], that is, children with a higher probability of presenting intellectual disability and associated medical conditions. The present text emphasizes the distinguishing features of the MIG program, discussing in depth the theoretical bases and evidence for the specific interventions, mainly motor, that make up the program. The text is divided into eight sections: clinical epidemiology of autism; biopsychosocial impacts of autism; neuropsychological profile of the population with autism; interventions for ASD; characterization of the Global Integration Method; flexible therapeutic suit based on myofascial rails (called MIG Flex) based on myofascial trains supporting complex motor synergies; evaluation of the results of the intervention; strengths and limitations and final considerations. MIG is an important step towards a more comprehensive and integrated approach to intervention. A salient characteristic of the proposed approach is that it adds interventions for sensorimotor issues based on newer concepts of neuropsychological mechanisms implicated in autism, i.e., the predictive coding and embodied cognitive perspectives (the definition of these and other medical and psychological terms is available in Supplementary Materials S1). As MIG integrates several new approaches from multiple disciplines, and considering space limitation intrinsic to a journal article, an appendix with recommended further readings is provided.

## 2. Clinical Epidemiology of Autism

Autism results from neurodevelopmental diversity, with onset in early childhood and diagnostic stability throughout the lifespan [11,12,13,14,15]. Autism manifests through widely varying behavioral characteristics in three domains of functioning: sociability, communication and environmental reactivity.

From a nosological point of view, autism can be characterized as a disorder or syndrome, with etiological and phenotypic heterogeneity [16]. Autism has become an important public health issue due to the increase in the number of diagnoses in recent decades. A recent meta-analysis showed the worldwide prevalence of ASD to be 0.6% (95% confidence interval: 0.4–1%) [17]. In school-age children, this prevalence is even higher, around 3% to 4% [18,19,20]. There is also evidence that the prevalence is 3 times greater in males [21]. The increase in the number of diagnoses may reflect both changes in diagnostic criteria and greater knowledge of autism, as well as a real increase in its incidence, probably caused by environmental influences [22,23,24,25].

The etiology of autism can be conceptualized in two large groups [26,27]. In a group of individuals, the etiology is more specific and identifiable, being associated with rare genetic variants with low familial recurrence or specific environmental agents. Many of the rare genetic variants are exemplified by genetic syndromes such as Rett, Cornelia de Lange, Tuberous Sclerosis, Angelman and Down syndromes, etc. [28]. Specific etiologies may also be environmental, exemplified by fetal alcohol and fetal cocaine syndromes [29,30,31,32,33], by maternal–fetal immune activation [29], or by exposure to high levels of fetal testosterone [34,35,36,37,38]. Cases with specific etiologies and syndromic characteristics correspond to 10–20% of the cases, and are more likely to present intellectual disability and less likely to have familial recurrence [26,27].

Around 80% to 90% of autism cases do not have a specific etiology, and these are characterized as idiopathic, probably multifactorial [24,26,27]. Cases of multifactorial etiology arising from the interaction between common genetic variants and environmental influences present a greater probability of familial recurrence or the occurrence of cases with an extended or milder autistic phenotype in the family. Multifactorial autism can be characterized as a psychological trait in itself, since it is not possible to identify a specific etiological agent [39,40,41,42].

The therapeutic challenge of autism is to meet the multiple demands imposed by the complexity of its manifestations and biopsychosocial impacts. This is discussed in the next section.

## 3. Biopsychosocial Impacts of Autism

**Figure 1:**
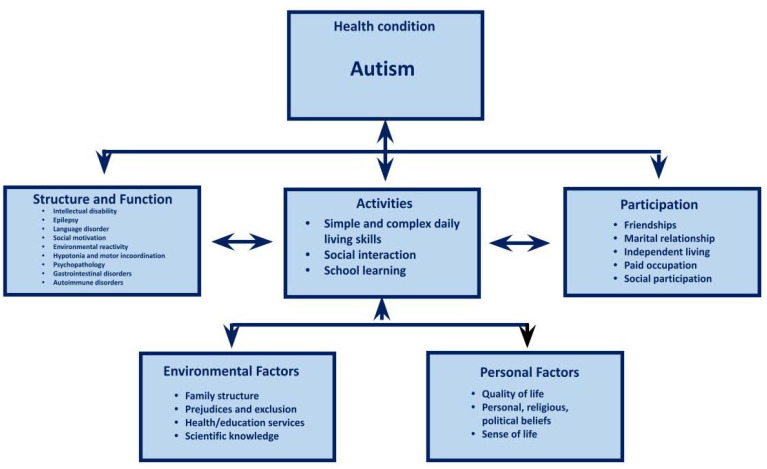
Biopsychosocial impacts of autism according to the WHO’s biopsychosocial model [46]. Explanation in the text.

The phenotypic variability in cases of both syndromic and non-syndromic autism is enormous, potentially expressed at all levels of impact of the World Health Organization’s (WHO) biopsychosocial health model [43,44,45,46] (Figure 1). The impacts on the five levels of the biopsychosocial model are described below.

### 3.1. Body Structure and Function

Dysfunctions in the epigenetic regulation of development and neuroplasticity allow us to understand not only the genesis of autism manifestations, but also the pattern of associated comorbidities. Current population estimates indicate that 30% of individuals with autism have intellectual disability [18,19,47]. However, the most recent estimates show that 25% to 30% of children with autism have borderline intelligence [18,19,48]. That is, around 55% to 60% of children with autism have cognitive changes which make their social and academic learning, as well as the acquisition of functional skills in daily life, difficult, constituting a risk factor for several associated conditions [49].

Difficulties with oral language are highly prevalent in autism. Fifty-seven percent of verbal children with autism present structural disorders in the acquisition of oral language [50], in addition to the social–pragmatic difficulties characteristic of autism [51]. Around 27% of children with autism do not acquire oral language skills [52,53]. Autism is associated with an increased risk of school learning disabilities, such as dyslexia and dyscalculia [54,55,56].

Motor manifestations in autism are a relatively neglected aspect [3], and there are few interdisciplinary and intensive models of autism care, including motor physical therapy [57]. Virtually all levels of the motor system are compromised in autism [58,59,60]. More than half of children with autism present hypotonia, and changes in balance, gait, appendicular motor coordination and motor planning are also common [61]. 

There is evidence that motor function is significantly associated with social skills in autism [58,59,60,62,63,64]. The knowledge about brain and neuropsychological development in the first year of life indicates that sensorimotor difficulties are among the first manifestations of autism, observable in a pre-clinical phase, suggesting that sensorimotor difficulties interfere with social interaction and can contribute to the development of autism manifestations [3,65,66,67,68]. The evidence indicates that there is basic instability in postural mechanisms in autism, which potentially interferes with socio-cognitive learning mechanisms. The understanding that early sensorimotor difficulties may play a role in the development of autism manifestations has been guiding the development of conceptual models of early, presymptomatic intervention [69]. One of the hallmarks of MIG is that it also intervenes in the motor difficulties of autism. This is discussed below.

The manifestations associated with autism at the body structure and function level illustrate the complexity of care demands, which need to be met by different professionals in the health and education areas. Impacts at the activity level are discussed below.

### 3.2. Activities

The cognitive, linguistic, motor and environmental reactivity peculiarities experienced by children with autism are expressed as difficulties at the activity level. The main difficulties are associated with atypicalities in social learning [13,70]. Autism is usually regarded as an atypicality in social learning, in that most of children’s learning occurs through interaction with adults and peers. Children with autism present difficulties in motivation for social contact as well as peculiar patterns of social reinforcement (e.g., smiles, hugs, speech, gestures), which make it difficult to acquire skills in the diverse situations of daily and social life [68].

In general, many children with autism may be characterized by: (a) apparent attenuated response to social stimuli (e.g., eye contact); (b) difficulty sharing attention and intention; (c) greater interest in objects than in people; (d) preference for solitary activities; (e) apparent reduced interest in social interaction; (f) reduction in initiative and maintenance of conversation; (g) manifest reduced pleasure from social interaction; (h) reduction in the search for social comfort; (i) difficulty in interpreting social stimuli; (j) peculiar reinforcers; (k) peculiar social interaction; etc. [11,12,13,14,15,26].

The social learning difficulties characteristic of autism also have repercussions at the activities level regarding the development of autonomy and functional occupational skills. In autistic children with intellectual disabilities, functional abilities are directly associated with IQ [71,72,73]. However, in autistic children without intellectual disabilities, the level of functional performance is around one standard deviation below IQ [74,75,76]. 

Next, the impacts of autism at the level of participation are discussed.

### 3.3. Participation

Participation may be impacted in autism in several ways. Children with autism tend to be lonely, facing difficulties in establishing and maintaining friendships [77,78]. Many children with autism do not have a clear idea of what friendship is, and the degree of intimacy and reciprocity involved [79,80]. Children with autism are infrequently mentioned in sociometric studies carried out in school classes, indicating a certain social “invisibility” [77]. Individuals with autism find it difficult to initiate and maintain sexual and marital relationships [81]. Furthermore, important restrictions on social participation, such as leisure activities, are observed. In adulthood, only 14% of individuals with autism are able to maintain a paid occupation and 49% need to live with their family [82]. The impacts of autism at the body structure and function and activities and participation levels are moderated by environmental and personal factors. Next, environmental factors are discussed.

### 3.4. Environmental Factors

Environmental factors can represent barriers or facilitators to the individual’s development. The main environments experienced by children with autism are the family, the clinics where they receive care and the schools.

#### 3.4.1. Family

While they depend on good family structure and functioning to develop, children with autism play a significant role in increasing family stress. Of all the neurodevelopmental disorders, autism has the greatest potential impact on the family [82,83,84,85,86,87,88,89,90,91,92]. Families having children with autism are more susceptible to disruption [93], and they present a lower quality of life [94,95,96] and a higher incidence of stress [92,97,98] as well as psychopathologies [99]. After the initial difficulties with the diagnosis, families face difficulties in obtaining the necessary therapies and in finding a school that meets their children’s needs. Difficulties in adolescence can be illustrated by the high incidence of gender dysphoria [100,101]. In adulthood, the family needs to make provisions to support persons with autism if they are unable to lead an independent life. Some autistic children present behavioral characteristics, such as strict adherence to routines, sensory reactivity, food selectivity, hyperactivity, sleep disorders, etc., that require such drastic accommodations from the family that the family becomes maladaptive [102,103]. The quality of health and education services directly affects the structure and well-being of families [94,95].

#### 3.4.2. Health Services

The complexity of the biopsychosocial impacts associated with autism requires a team of multiple health professionals. Treatments need to start early and to be intensive. The costs associated with treatments are enormous, not only from a financial point of view but also in terms of time, energy and affection [103,104]. Multiple treatments carried out by different professionals working independently or maintaining sporadic contact are extremely exhausting. There is also a risk that different professionals act in conflicting manners, pursuing different objectives. Ideally, health services should be provided in an interdisciplinary manner by a coordinated team, giving opportunities for the family to actively participate. Family participation may occur in the decision-making process or in acting as co-therapists and generalizing therapeutic practices from the clinic to the home environment [105,106]. Access to healthcare is associated with quality of life for families of children with autism [96,107].

#### 3.4.3. Educational Services

Attending regular schools and classes is an ideal and a right, hard won by families with children with autism over decades of political mobilization [14]. However, the results of inclusive education are still far from what is desired [14,108]. Most children with autism have difficulty following the curriculum, and schools have difficulty making the necessary curricular adaptations. Often, schools emphasize an academic curriculum that is beyond the ability of many children with autism, while neglecting a functional curriculum that promotes autonomy and independence [14]. The difficulties encountered with inclusion in regular classes have sparked interest in alternative models. As children with autism have difficulties with social learning, imitation and collaboration in groups [13,109], simply being together with typical peers is not enough to promote their learning and social inclusion. Interdisciplinary interventions are necessary to make inclusion effective. 

The discussion about the impacts of autism at the environmental factors level demonstrates the complexity of the health and educational needs associated with autism. Next, impacts at the personal factors level are discussed.

### 3.5. Personal Factors

People with autism have reduced perceptions of quality of life compared to typical individuals [6]. Their perceptions of quality of life reflect the multiple impacts associated with autism, mainly with participation [110]. Individual characteristics, such as intelligence and beliefs (personal, religious and political), constitute important moderators of the perceptions of quality of life in individuals with autism [111]. Difficulties are worsened by loneliness and lack of financial and decision-making autonomy [78,79,80,81,82]. In addition to other psychiatric problems, the risk of suicide is high in persons with autism [112,113]. In Brazil, practically the only community resources available after the age of 18 are support networks for people with intellectual disabilities or multiple disabilities, such as the Associação de Pais e Amigos dos Excepcionais (APAE) [114].

## 4. Neuropsychological Profile of the Population with Autism

The profound epidemiological changes that have occurred in recent decades have caused a change in the neuropsychological profile of the population with autism. As the prevalence of autism increased, the prevalence of people with autism and intellectual disabilities decreased from around 70% to 30% [18,19,47]. At the same time that this was occurring, the magnitudes of effects of neuropsychological markers that differentiated samples of individuals with and without autism decreased from up to two standard deviations to fewer than 0.5 standard deviations in many cases [115]. This suggests that the increase in prevalence is at least partially related to the increasing identification of autistic traits in individuals without intellectual disability.

The change in the neuropsychological profile of individuals with autism may partially explain the emergence of a new perspective, the identity theory of autism as neurodiversity [12,14]. This perspective proposes that: (a) autism should be considered as a collective identity; (b) autism is not related to any pathology, but simply to a different type of brain, constituting a diversity that characterizes a neurominority; (c) the autistic neurominority is socially and politically stigmatized and oppressed; (d) as it is an identity, it is most appropriate to refer to autistic people not to people “with autism”, since this reifies and makes autism more normal; (e) autism needs to be defined in terms of its advantages and not in terms of deficits, contributing to the development of autistic pride; (f) autism is a valid mode of existence that needs to be recognized and to which society and not the autistic individual needs to adapt; (g) education should not be “special” or “inclusive”, but rather accessible; (h) self-identification as autistic takes precedence over diagnosis by specialists; (i) as there is no disease or deficiency to be “cured”, the neurodiversity identity movement discourages research into neurogenetic bases; and (j) as individuals with autism have the right to self-determination and as, strictly speaking, only they have authority to speak, their participation in the formulation of public discourse and policies must be predominant.

A neurodiverse perspective certainly meets the rights and needs of a growing contingent of individuals with autism, especially adults with higher intelligence or requiring level 1 support in DSM-5 terminology [1]. However, it may conflict with the rights and needs of individuals with associated patterns of comorbidities, such as intellectual disabilities and genetic syndromes, corresponding to levels 2 and 3 of support. It is also necessary to consider that: (a) although the number of people with autism and normal intelligence has substantially increased in recent years, around 55% of children with autism in demographic representative samples have an IQ below 85 [48]; (b) the variability in the prevalence of autism diagnosis without intellectual disability from one research center to another is three times greater than the variability in the prevalence of autism diagnosis with intellectual disability [18,19], indicating that the diagnosis of autism without intellectual disability may be more complex and with lower inter-examiner reliability; (c) studies with clinical samples show a higher prevalence (around 50% to 55%) of intellectual disability in autism [21,116] compared to a prevalence of 30% in population samples [18,19,47], indicating that children with autism associated with intellectual disability may have greater or different assistance needs; (d) a sampling bias towards high intelligence was identified in research published in leading journals since 94% of participants did not present intellectual disability [117], suggesting that the needs for research on autism in association with intellectual disability may not be receiving due consideration; and, finally, (e) a fourfold increase in the risk of dementia occurring between 30 and 64 years of age has been identified for individuals with autism compared to the general population [110], indicating that persons with autism are neurologically vulnerable and require specific healthcare. MIG was designed considering the health and education needs of more vulnerable individuals with autism.

Phenotypic heterogeneity is the hallmark of autism. It has been notoriously difficult to characterize endophenotypes that bridge between the multifactorial etiologic influences and the autistic phenotype [16]. Evidence is emerging, however, that at least two fuzzy prototypes may be identified [26,27]. On the one hand, a growing contingent of individuals with autism do not present intellectual disability. In these cases, there is a stronger preponderance of occurrence in males, the family recurrence of the broad phenotype is higher, genetic markers are represented by common alleles, the association with parental age is low, there is an association with longer intergestational intervals and macroencephaly is more frequent. On the other hand, some individuals with autism present intellectual disability or some degree of cognitive change. In these cases, there is a weaker preponderance of occurrence in males, the family recurrence of the broad phenotype is lower, genetic markers are represented by rare alleles or new mutations, the association with parental age is higher, there is an association with shorter intergestational intervals and brain size is variable. These two prototypes may represent extremes of a continuum, reflecting the heterogeneity of the autistic phenotype. An intervention program for autism must consider the entire, or at least the largest possible, range of phenotypic variations, meeting the needs of those children with more severe conditions, including those associated with intellectual disabilities and/or genetic syndromes. The price to be paid may be the irrelevance or even offensiveness of this approach for people with autism who are cognitively more fortunate.

## 5. Interventions for ASD

The last few decades have witnessed remarkable progress in interventions for autism, with cumulative evidence regarding their validity, especially studies with single-subject experimental designs. Most of these interventions are based on the hypothesis that autism consists primarily of an atypicality in social learning. In general, four trends can be identified in the literature. The first studies evaluated applied behavior analysis in relatively limited clinical settings using discrete trial training (DTT). DTT has been used successfully both to manage behavior problems and to develop abilities in preschool children participating in intensive intervention programs ranging from 20 to 40 h/week [118]. Behavioral principles have also been applied in the school setting using the TEACCH approach [9]. More recently, the behavioral approach has benefited from integration with evidence on the trajectory of children’s social development in naturalistic settings. This has given rise to naturalistic developmental behavioral interventions (NDBIs) such as the Early Start Denver Model [119], Joint Attention, Symbolic Play, Engagement and Regulation (JASPER [120]) and Pivotal Response Training [121]. Criteria for establishing intervention validity have been established [122]. As these programs require intensive intervention for several hours per week, the costs are extremely high. One way to reduce costs, while maintaining the treatment intensity, is to work with parents as co-therapists. This has been proposed in programs such as Project ImPACT (Improving Parents as Communication Teachers [123]). Working with parents as co-therapists may eventually improve generalization as the treatment is implemented in the home.

While it is important to recognize the effectiveness of behavioral programs, it is also necessary to recognize their limitations. Behavioral interventions for autism focus on maladaptive behaviors and the development of social communication abilities. An important phenotypic dimension of autism, consisting of impairments of environmental reactivity such as stereotypies, restricted repertoires of behaviors and interests, resistance to change and insistence on sameness, hypo- and/or hyper-sensory reactivity, anxiety symptoms, etc., remains unaddressed or unexplained.

As will be seen in the next section, recent theoretical developments related to the predictive coding deficit hypothesis and embodied cognition impairments have important explanatory and intervention implications.

## 6. Global Integration Method (Método de Integração Global–MIG)

Considerations of the complexity and potential severity of the biopsychosocial impacts associated with autism support the need for integrative and intensive intervention programs, which should begin as early as possible [124]. These programs should be delivered in an interdisciplinary manner by a well-trained and cohesive team [105,106], working in a single location and in concert with the family in the pursuit of common goals [125,126]. 

The MIG does not consist of just a single intervention method, but rather an integrated and interdisciplinary program of multiple intervention strategies which seek to consider the greatest possible number of levels of impact, according to the WHO’s biopsychosocial model and its complex interactions [48]. The main characteristics of MIG, its theoretical–methodological foundation and the respective references are described in Table 1.

Table 2 describes the units of the City of Tomorrow, a naturalistic environment where many of the activities are developed with the child. The City of Tomorrow is an immersive therapeutic space that provides greater involvement of children and adolescents, has reward systems (based on applied behavior analysis [148]) and uses structured cognitive models of learning (based on the theory of cognitive overload, which bases learning on the development of cognitive schemes [138,139]). The City of Tomorrow is composed of units called naturalistic learning environments, in which the child has the opportunity to carry out activities relevant to life in situations that resemble their natural environment (i.e., home and school) in a playful way and using concrete materials.

In each City of Tomorrow unit, children have the opportunity to develop intrinsically reinforcing activities while developing powerful cognitive schemes, and learning to use them in a concrete and contextualized way that is relevant to life. The use of cognitive schemas is based on the cognitive load theory [138,139]. Working memory constitutes the locus of learning [181]. As processing capacity is limited in working memory, overload needs to be avoided for any learning to take place. The construction of complex knowledge structures called schemas contributes to information processing by reducing working memory overload [139].

The main cognitive schemes worked on and which underlie the City of Tomorrow units are: (a) social information processing theory [148,163]; (b) self-instruction in problem solving [155,156,157]; (c) story grammar [168,169,170,171]; (d) autobiographical memory [154,155,170]; (e) trajectory model of number concept development [161,162]; (f) cognitive–neuropsychological models of oral lexical processing [164,167] and the reading and writing of words [165,167] and numbers [166,182]; and (g) cognitive–neuropsychological models of drawing [172,175].

The use of cognitive schemes in the City of Tomorrow can be illustrated through the Supermarket (“Mercadinho”) Unit. The Supermarket Unit simulates two important scripts of daily life: going to the supermarket and a children’s party. The children receive a visit from a little Martian friend and plan to have a welcome party for which they need to go shopping at the supermarket. Children are invited to reflect on the scripts for going to the supermarket and organizing parties, explaining how everything works to another child without any prior experience. The visit to the Supermarket offers the opportunity to reflect on the causal–temporal structure of these itineraries, on the essential and accessory components, etc. At the same time, the child can develop vocabulary and functional and taxonomic categorization skills of items available in the supermarket, as well as develop elementary numerical–arithmetic skills related to quantities, numerical representations and operations with small sets. Planning skills are developed through the self-instruction schema for problem solving [156,158].

Some children with neurodevelopmental disorders present more serious difficulties in organizing their behavior and attention. Problems with executive functioning are exemplified by difficulties in concentrating, sustaining and dividing attention; maintaining and processing information in working memory; inhibiting inappropriate responses to the current context; and planning to monitor and implement problem-solving strategies flexibly that allow behavior to be organized according to objectives, etc. [183]. These difficulties are accentuated in children with intellectual disabilities, autism and attention deficit hyperactivity disorder. The Intuitive Learning Desk, which is used to work on attention and executive function and to develop work routines, aims to offer physical support that enables the organization of behavior to promote more effective learning for children with the greatest need for support. 

**Figure 2:**
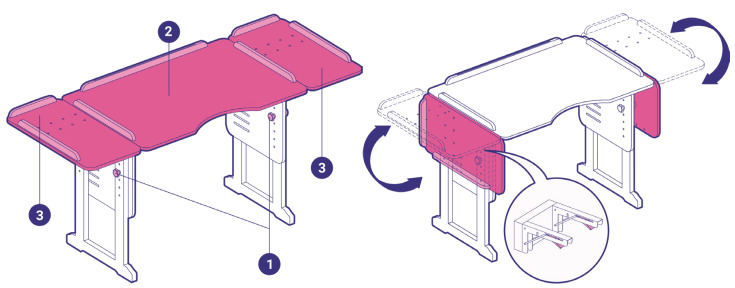
The Intuitive Learning Desk. Legend: 1 = handles for height (allowing height variation from 52 to 70.7 cm) and inclination adjustment; 2 = task execution area (75 × 50 cm); 3 = side flaps (47 × 33 cm each).

The desk has raised edges to make it easier to understand the division of the space, as well as to prevent materials from falling to the floor. The desk allows height and inclination adjustment, and is spatially organized into a central top (task execution) area with a concave cut at the bottom, which allows the student to be closer to the desk, and two foldable side flaps. The divisions “Left Side Flap”, “Execution Area” and “Right Side Flap” allude to the processes of starting, executing and ending the proposed task, respectively (Figure 2). Tasks are offered and performed from left to right, just like the Western reading and writing pattern.

In the next section, the basis of MIG in evidence is discussed.

### 6.1. Evidence Rationale for MIG

MIG is a complex program of interventions with the following hallmarks: (a) intensive interdisciplinary care by a single team working in an integrated manner in a single location; (b) coverage of all impact levels of the International Classification of Functioning, Disability and Health (ICF–Body Structure and Function; Activities and Participation; Environmental and Personal Factors); (c) specific physical therapeutic and cognitive interventions for socio-motor manifestations, using both bottom-up (flexible therapeutic suit based on myofascial trails and development of cognitive schemas) and top-down (socio-motor synchronization, motor planning through imagery, self-instruction in problem solving, etc.) strategies; (d) decisions shared with the family, and parents acting as co-therapists in managing behavioral problems and promoting social learning skills; (e) manualization, continuing training and supervision of the professionals involved; and (f) management through application.

MIG was developed from a broad literature review of original and secondary research (systematic reviews and meta-analyses) [2,184,185,186,187]. Each of the MIG components is supported by empirical evidence, presented in Table 1. Each of the characteristics and interventions that compose MIG was selected based on the availability of evidence regarding its effectiveness. Established clinical guidelines, results of original empirical studies, systematic reviews and meta-analyses from randomized, controlled clinical trials as well as quasi-experimental studies with single-subject designs were considered. Each of the components in Table 1 is based on an empirical–theoretical–methodological rationale and available evidence [25,184,185]. The strength of the MIG is that it integrates all these components into a comprehensive program, implemented by professionals who are trained and supervised. The principles of applied behavior analysis embodied by MIG are described below.

### 6.2. Applied Behavior Analysis

In the 1970s, Ole Ivar Lovaas began a series of studies of interventions based on applied behavior analysis for individuals with autism, finding favorable results for the practice [188,189]. Since then, other intervention modalities based on applied behavior analysis (ABA), such as discrete trial training (DTT), pivotal response treatment and naturalistic and developmental behavioral interventions (NDBIs) have been developed. 

The most widespread practices using evidence-based interventions for ASD are based on ABA [2], which is considered a science in itself and not just a method or type of intervention. ABA is the science that applies knowledge from the assumptions of Skinner’s radical behaviorism to contribute to issues of social relevance, such as education and health [190]. The method developed by Lovaas [118,188,189], based on applied behavior analysis, is known as DTT. 

An important advancement in the psychological therapies for autism was the advent of naturalistic, developmental behavioral interventions, or NDBIs [8,9,10,11,12,13,14,15,16,17,18,19,20,21,22,23,24,25,26,27,28,29,30,31,32,33,34,35,36,37,38,39,40,41,42,43,44,45,46,47,48,49,50,51,52,53,54,55,56,57,58,59,60,61,62,63,64,65,66,67,68,69,70,71,72,73,74,75,76,77,78,79,80,81,82,83,84,85,86,87,88,89,90,91,92,93,94,95,96,97,98,99,100,101,102,103,104,105,106,107,108,109,110,111,112,113,114,115,116,117,118,119,120,121,122,123]. NDBI programs add naturalistic and developmental considerations to the traditional behavioral methods developed under ABA. The naturalistic–developmental behavioral approach emphasizes the involvement of parents as co-therapists [8,9,10,11,12,13,14,15,16,17,18,19,20,21,22,23,24,25,26,27,28,29,30,31,32,33,34,35,36,37,38,39,40,41,42,43,44,45,46,47,48,49,50,51,52,53,54,55,56,57,58,59,60,61,62,63,64,65,66,67,68,69,70,71,72,73,74,75,76,77,78,79,80,81,82,83,84,85,86,87,88,89,90,91,92,93,94,95,96,97,98,99,100,101,102,103,104,105,106,107,108,109,110,111,112,113,114,115,116,117,118,119,120,121,122,123]. MIG follows this trend in that it emphasizes activities in naturalistic environments with relevance to practical and social everyday life, as well as parental involvement. MIG also uses conceptual tools from cognitive psychology [140,141] based on the assumption that children with autism have difficulties with anticipating events, which originates from inaccurate predictive models, rigidities or difficulties in handling prediction errors [191]. This will be discussed in the Relevance of Predictive Coding for Autism section. 

MIG is characterized as an interdisciplinary intervention proposal for ASD, based not only on the principles of ABA science, but also on principles of cognitive neuropsychology and evidence-based practices, such as those mentioned above. The concepts and techniques of behavior analysis are implemented in MIG through a double strategy. On the one hand, professionals from different specialties are trained to use naturalistic–developmental behavioral strategies. The various therapeutic activities provide an opportunity for professionals to manage inappropriate behaviors in a non-coercive way and to develop prosocial and occupational skills in the child. This is implemented following the child’s lead through a playful and narrative context in a naturalistic environment, the City of Tomorrow. The strategies are based on differential reinforcement with natural reinforcers, shaping and chaining with cues and cue fading, organization of the environment and structuring of routines, child-initiated learning, shared control and reciprocity in activities, expansion of attentional focus, playful imitation of the child by the adult, modeling and managing the level of stress and emotions, etc. [8]. On the other hand, parents receive one hour per week of individualized counseling on parent training based on the same principles [123]. At least three parent training curricula are offered, each lasting 15 weeks. In the first semester, parents learn the basics of child social development and applied behavior analysis, learning how to use non-coercive problem management strategies. In the second semester, parents work on the development of social skills (shared attention, imitation, play, shared intention, etc.) and occupational skills (nutrition, personal hygiene, etc.) according to need. From the third semester of intervention, families begin to receive individualized parent training programs, focusing on specific issues on demand.

Therefore, the program proposes an intensive and comprehensive treatment covering a wide range of characteristics associated with autism, going beyond difficulties in socio-communication and encompassing sensorimotor aspects, language and daily life skills, among others.

MIG recommends that the evidence base for non-pharmacological interventions for autism not depend only on the application of Protocol A or B, but that it include the clinical reasoning of professionals working as a team and collaborating with families in the development and monitoring of therapeutic goals. Decisions need to be made as a team, working together with the family to establish goals, formulate hypotheses regarding the causes of certain problems and systematically test these hypotheses through specific interventions. The evidence-based healthcare philosophy underlying MIG can only be implemented at the expense of the ongoing training and supervision of the professionals involved.

Having thus far discussed the characteristics of the main components of MIG and its evidence base, the following sections discuss the theoretical–methodological foundation of one of the main hallmarks of MIG, precisely the interventions at the body structure and function level, which are mainly related to motor manifestations. The evidentiary basis for this approach comes from evidence regarding the importance that predictive coding dysfunctions play in autism [15,191] and the embodied basis of socio-cognition [192]. First, however, the traditional psychological approach that considers autism basically as a social learning atypicality is discussed.

### 6.3. Relevance of Social Learning for Autism

According to Siegel (2018) [14], autism can be considered a learning disorder comprising three important domains: (a) social interaction, (b) communication and (c) environmental reactivity. Social learning is essential for a child’s cognitive, communicational and affective development. Children learn from observing the consequences of their interactions with others and from observing the behavior of others in different contexts, through direct and vicarious learning mechanisms.

Many children with autism pay more attention to details and objects than to social situations and people, presenting difficulties in learning from interaction. Children with autism tend to be more responsive to instrumental reinforcement than to social reinforcement. Children with autism experience difficulties with cognitively mediated imitation, as well as with symbolic and social play, which are essential for development and learning.

The most effective intervention approaches for autism are based on applied behavior analysis, seeking to make the child more sensitive to social reinforcement and using social reinforcement to develop skills of shared attention and intention, cognitively mediated imitation and symbolic and social play [8].

One of the limitations of more traditional theories and interventions for autism is their substantial focus on the socio-communicational dimension to the detriment of environmental reactivity [15,193]. An important group of autism traits relates to environmental reactivity: (a) children with autism tend to be hyper- and hypo-reactive to sensory stimulation; (b) children with autism tend to self-stimulate and present stereotypies as a way of controlling their level of excitability; (c) children with autism tend to present neophobia, adhering rigidly to routines and showing resistance to change; (d) children with autism tend to show hyperfocus of attention on details, restricting their repertoire of interests and behaviors; and (e) children with autism tend to present instability in motor and postural regulation mechanisms, which manifests as excessive micromovement, difficulties with static and dynamic balance and difficulties with appendicular coordination and motor planning.

Two alternative theoretical approaches, based on the ideas of predictive coding [15,191] and embodied cognition [192], allow for an understanding of both the socio-communicational difficulties and the environmental reactivity observed in autism. Theories of deficits in predictive coding and embodied cognition in autism underlie the use of flexible therapeutic suits based on myofascial trails, which constitute a hallmark of MIG. This is discussed in the next two sections.

### 6.4. Relevance of Predictive Coding for Autism

The social learning deficit hypothesis does not adequately explain symptoms related to atypicalities in environmental reactivity [13]. A hypothesis with the potential to explain the three dimensions of autism (social, communicational and reactivity) is that the basic difficulty in autism may be related to a deficit in predictive coding [15,194]. The acronym PIA, derived from “prediction impairment in autism”, was proposed to refer to this hypothesis.

The predictive coding hypothesis originates from considerations that the brain processes and interprets sensory information so that it can be used to construct the phenomenological experience and regulate the decision-making processes [15,194]. The brain constantly builds predictions concerning the state of the world and seeks to minimize any discrepancies between these predictions and sensory inputs.

The brain is structured hierarchically [194]. The lower levels of the hierarchy represent specific details of the sensory input. The higher levels of the hierarchy represent interpretations at increasing levels of abstraction and generalization. At each level of the hierarchy, predictions are generated about the nature of the sensory input based on previously acquired experiences and knowledge. A discrepancy between predictions and sensory input constitutes a predictive error, and the main objective of hierarchical brain organization is to minimize predictive errors. Minimizing errors can occur through updating predictions, building new internal models (schemas) or through action to transform reality so that sensory inputs align with predictions.

Predictions represent top-down processing mechanisms, that is, a descending flow of control [194]. Prediction errors constitute bottom-up processing mechanisms, that is, an upward flow of control. The learning process consists of updating internal models or schemas based on prediction errors. The brain learns from its mistakes, adapts to changes and is able to make increasingly more credible predictions. The construction of internal models or schemes is based on bottom-up mechanisms of statistical learning, or Bayesian inference [193]. 

Predictive coding in autism can be compromised in at least three main ways: (a) deficits in the statistical learning processes in the form of difficulties with prototypical categorization and formation of cognitive schemes, resulting in poorly predictive internal models; (b) deficits in the statistical learning processes or neglect of context, resulting in the construction of rigid and inflexible internal models; and (c) deficits in the descending control mechanisms, corresponding to the impairments in executive functioning observed in autism. Empirical evidence is still insufficient to decide among these alternatives [191]. Most likely, these three hypotheses are complementary.

The hypothesis that deficits in predictive coding occur in autism has important implications for intervention. The emphasis on autism adopted in MIG is justified by the demographic prominence of this condition. At the same time, the implications of the predictive coding hypothesis are important not only for autism, but also for other conditions such as intellectual disability, ADHD, anxiety and even lack of stimulation. The main intervention strategies basically consist of explicit instruction based on error-free learning to develop more credible and flexible internal predictive models, involving the child in playful and concrete activities in a naturalistic environment. Both bottom-up and top-down strategies are used.

The bottom-up strategy consists of providing experiences and reflection that encourage the development of cognitive schemas, which give meaning to subjective and objective reality. Narratives constitute one of the most powerful cognitive schemas, as they allow for thematizing of conceptual, factual, procedural, social and practical aspects of learning. In this context, strategies based on feedback from the brain to the lower-lying parts of the brain hierarchy (top-down strategies) are important to allow the brain to check the value of its own predictions about the environment. In other words, information originating from senses is sent to the brain, and then the brain responds by selecting the information that is useful for its comprehension of the environment. Thus, an important strategy is to reduce processing overload in working memory, and consequently, more resources are available for processing novelty and contextual idiosyncrasies. 

Autism presents motor manifestations at multiple levels. There are also manifestations of the axial and appendicular muscles, computational mechanisms (planning, feed-forward control and execution), functional mechanisms (e.g., intention, goal, neuromotricity, kinematics), the level of neural integration (e.g., cortex, basal ganglia, cerebellum), etc. The motor skills of people with autism may be compromised at different levels. At a very basic level, autistic people present instability in postural mechanisms, which manifests itself in the form of exaggerated micro-movements. Most likely, the exaggeration of micro-movements is related to a deficit in reafference mechanisms (efferent copies or corollary discharges) [195].

Evidence suggests that motor alterations may play an important role in the development of social skills in autism [4,58]. The two main mechanisms implicated are resonance and interference [58]. The mirror neuron system is one of the neural mechanisms enabling the activity of an individual’s neuromotor system to resonate with the motor activity of other individuals. This socio-motor resonance is essential for all psychological, social, cognitive, socio-emotional, etc., development. Resonance is a computational property of complex systems, such as the brain, that function in an oscillatory way. Whenever two systems come into oscillatory-phase synchrony, they resonate. That is, phase transitions, i.e., qualitative changes in information processing and development, occur. Individuals with autism have difficulties with motor synchronization mechanisms. In a finger tapping task, the phase anticipation of autistic people is always greater. This difficulty is not related to an intrinsic deficit in the internal generation of rhythms, but rather to a difficulty in synchronization caused by detecting invariance in the environment. Evidence suggests difficulties with socio-motor synchronization. 

Socio-motor synchronization is essential for social, affective, cognitive, etc., development. These psychomotor synchronizations begin in the womb and continue in the baby’s first bodily interactions with the mother. Difficulties in socio-motor synchronization in autism are complicated by the fact that difficulties in statistical learning and in detecting invariances lead to a deficit in detecting biological motion. If typical people need to make a gesture at the same time as they watch another person making the opposite gesture, a cognitive conflict arises and people become confused. Autistic people do not experience this conflict because they are less affected by “biological movement”. Autistic people may find socio-motor synchronization easier with robots [196,197]. The other mechanism by which motor changes influence socio-cognition is mutual interference, discussed in the next section on the relevance of embodied cognition for autism.

### 6.5. Relevance of Embodied Cognition for Autism

Embodied cognition considers that mental activity in general, and cognitive activity in particular, have origins in bodily action mechanisms [192]. Illustrative examples of the embodied origin of cognitive activity are verbal working memory and the development of syntax and arithmetic skills.

The first arithmetic activity performed by children is counting. The use of fingers to count and solve arithmetic operations during a child’s development is practically universal. Finger counting plays the important role of reducing the processing burden on working memory as the child learns to coordinate the dual tasks of reciting the number series and making the correspondence between the recited numbers and the objects counted [198]. Over time, children begin to count without using their fingers, counting only verbally until they develop conceptual and mnemonic problem-solving strategies.

The examples above illustrate how bodily sensorimotor activity forms the foundation for the development of progressively more abstract cognitive skills. Most children with neurodevelopmental disorders present sensorimotor changes [199], ranging from basic disorders of postural tone reflected in excessive micro-movements to changes in static and dynamic balance and various forms of appendicular coordination [135].

Sensorimotor instability consumes energy and information processing resources, which are scarce [135]. Motor action and social interaction share internal regulatory mechanisms [137]. Therefore, the energy and attention that the child spends to stabilize the motor system drains resources that should be applied to other areas of learning, such as sociability and cognition. This creates a dual-task situation in which the need for voluntary control over sensorimotor stabilization competes with socio-cognitive activities related to learning for processing resources [130,132,134,136].

Sensorimotor action is fundamental for affective and social development [68,200]. The exchange of affect, as well as social interaction in general, depend on different forms of synchronization of an individual’s bodily activity, such as gaze, with the activities of other people [200]. Sensorimotor interactions compromised early in autism [201] are important precursors of language and socio-emotional development [202,203,204], constituting therapeutic targets of naturalistic–developmental behavioral interventions [2,8,9,10,11,12,13,14,15,16,17,18,19,20,21,22,23,24,25,26,27,28,29,30,31,32,33,34,35,36,37,38,39,40,41,42,43,44,45,46,47,48,49,50,51,52,53,54,55,56,57,58,59,60,61,62,63,64,65,66,67,68,69,70,71,72,73,74,75,76,77,78,79,80,81,82,83,84,85,86,87,88,89,90,91,92,93,94,95,96,97,98,99,100,101,102,103,104,105,106,107,108,109,110,111,112,113,114,115,116,117,118,119,120,121,122,123,124,125,126,127,128,129,130,131,132,133,134,135,136,137,138,139,140,141,142,143,144,145,146,147,148,149,150,151,152,153,154,155,156,157,158,159,160,161,162,163,164,165,166,167,168,169,170,171,172,173,174,175,176,177,178,179,180,181,182,183,184,185,186,187,188,189,190,191,192,193,194,195,196,197,198,199,200,201,202,203,204]. Use of MIG Flex is based on the empirical–theoretical assumption that motor stabilization facilitates social, emotional and cognitive development.

Bidirectional interference between motor and cognitive activities occurs in dual-task paradigms, such as bouncing a ball (or walking) or solving a math problem at the same time [132]. Motor–cognitive interference is frequently observed in the elderly [205]; in neurological diseases, such as multiple sclerosis [206]; and in neurodevelopmental conditions such as Down syndrome [130], cerebral palsy [133] and autism [131,133]. The individual needs to spend scarce working memory resources to regulate motor activity. As these processing resources are limited, there are fewer resources left to process socio-cognitive information. If the individual is absorbed with maintaining their posture, they are less able to pay attention and absorb what is happening in their environment, especially their social environment, which is the most dynamic. MIG Flex, which is based on dynamic myofascial geometry, fulfills the dual purpose of: (a) reducing the effects of interference between motor and socio-cognitive activity and (b) providing opportunities for the child to acquire control over their physical and social environment through activities in which they learn to regulate and use body action for exploring visual space, for recognizing and using objects, and for social interaction. The next section discusses the theoretical foundation of MIG Flex.

## 7. Flexible Therapeutic Suit (MIG Flex) Based on Myofascial Trains

Different technologies have been developed to enhance the results of rehabilitation for children with developmental disorders. Among these technological innovations, for example, therapeutic suits are very popular in different countries and with various models aiming at promoting improvement in gross motor and postural function, increasing speed and gait cadence and improving trunk control [205,207,208].

The therapeutic suits were inspired by the “Penguin Suit” developed for the Soviet space program in the late 1960s [209]. This first model of the suit was designed to offer resistance to body movements and promote tissue and biomechanical adaptation of astronauts in conditions of reduced gravity in space, minimize adverse effects such as muscular atrophy and osteopenia and maintain neuromuscular fitness [209]. Following this, other suits were developed to be associated with intensive and specific treatment protocols for neurofunctional rehabilitation, for example, the Adeli Suit^®^, the TheraSuit^®^ and the PediaSuit^®^. These models have hooks that anchor a system of individually fixed elastic tubes to exert traction between the trunk and the pelvis and between the pelvis and the lower limbs. The action mechanism, proposed to explain the effects provided by these therapeutic suits, is that of continuous compression exerted by the elastic elements of the clothing on the child’s musculoskeletal system [210].

However, the mechanical model of structural stability, based on continuous compressive forces, has limitations in terms of explaining the stability of joints in the human body [211]. Given the limitations of compressive models to explain the inherent stability of the musculoskeletal system, the tensional integrity (tensegrity) model was proposed [211,212,213]. A tensegrity system consists of an intrinsically stable system that contains a group of components in compression, within a network of interconnected components under continuous tension [212]. Applying this model to the musculoskeletal system, the bones represent the discontinuous compressive elements, and the soft tissues—fascia, ligaments, muscles and tendons—form the continuous network of tensional elements in which the bones are intrinsically connected [212,213]. It is believed that the organization of the musculoskeletal system is consistent with that of tensegrity structures, with the pre-stress present in the connective tissues of the musculoskeletal system.

The study of these continuous networks of tensional elements began in the 1940s, with different studies on “muscle chains” [214]. Based on this concept of muscle chains, and from the perspective of the tensegrity model, Myers (2001) and Stecco (2006, 2009) [215,216,217] describe models explaining “trains” that comprise the myofascial connections that cross the entire body. These “trains” are based not only on functional connections (as described by muscle chains), but also on anatomic connections between muscles and fascial tissue. The authors further postulate that these “trains” are involved directly in the organization of movement and in the transmission of muscular force [215,216,217,218,219]. Furthermore, the study on the innervation of fascial tissue also demonstrates its role in proprioception [220].

Fascial tissue can be divided into superficial fascia, visceral fascia and deep fascia, with the latter subdivided into aponeurotic fascia and epimysial fascia [221]. With the development of new studies and methodological advances in research, it has been seen that different fasciae have different types of innervations [220,221,222,223]. The superficial fascia has mechanical and thermal receptors that play an important role in thermoregulation, exteroception and pain perception [222,223]. The two types of deep fascia, aponeurotic and epimysial, have distinct mechanical functions and mechanisms. The aponeurotic fascia surrounds several muscles, holds them in place and connects them. The epimysial fascia is specific to each muscle and is tightly attached to them, defining their shape and volume. Histological studies on the deep fascia (in animals and humans), in turn, show its role in proprioception, with neuromuscular spindles and Golgi tendon organs observed in tissue analyses [220,223]. Furthermore, the connection between the capsule of the neuromuscular spindles and the connective tissue has been verified, which means that the function of the neuromuscular spindles has shown a direct relationship with the quality of the intramuscular and surrounding connective tissue [150]. Thus, changes in myofascial tissue can distort the information sent by the spindles to the central nervous system and, therefore, can interfere with the appropriate regulation of muscle tone and the quality of movements performed.

In addition to being innovative regarding neurodevelopmental disorders, this theoretical foundation is extremely important for the development of interventions and technologies for people with autism. This is because the correct planning and execution of movements require both a correct efferent motor stimulus and good motor control, based on appropriate proprioceptive stimuli. The MIG Flex was developed based on myofascial lines and general geometric patterns, also considering the changes in motor control, feedforward and feedback, as well as muscular hypotonia presented by children with autism [224,225] and the role of the quality of myofascial tissue on feedforward and feedback to the central nervous system, leading to the regulation of muscle tone.

Based on the above, the MIG Flex is based on the dynamic myofascial lines [149] with structural frameworks that simultaneously allow for stabilization, movement and body perception. Myofascial geometry is composed of rails that connect the different layers of connective tissue and muscle fibers of the body, including the fascia, aponeurosis, tendons and muscles. The transmission of tension through the myofascial rails reproduced by the MIG Flex allows for the implementation of more complex motor synergies, integrating different joints and segments of the body in a complex, organized gesture.

**Figure 3:**
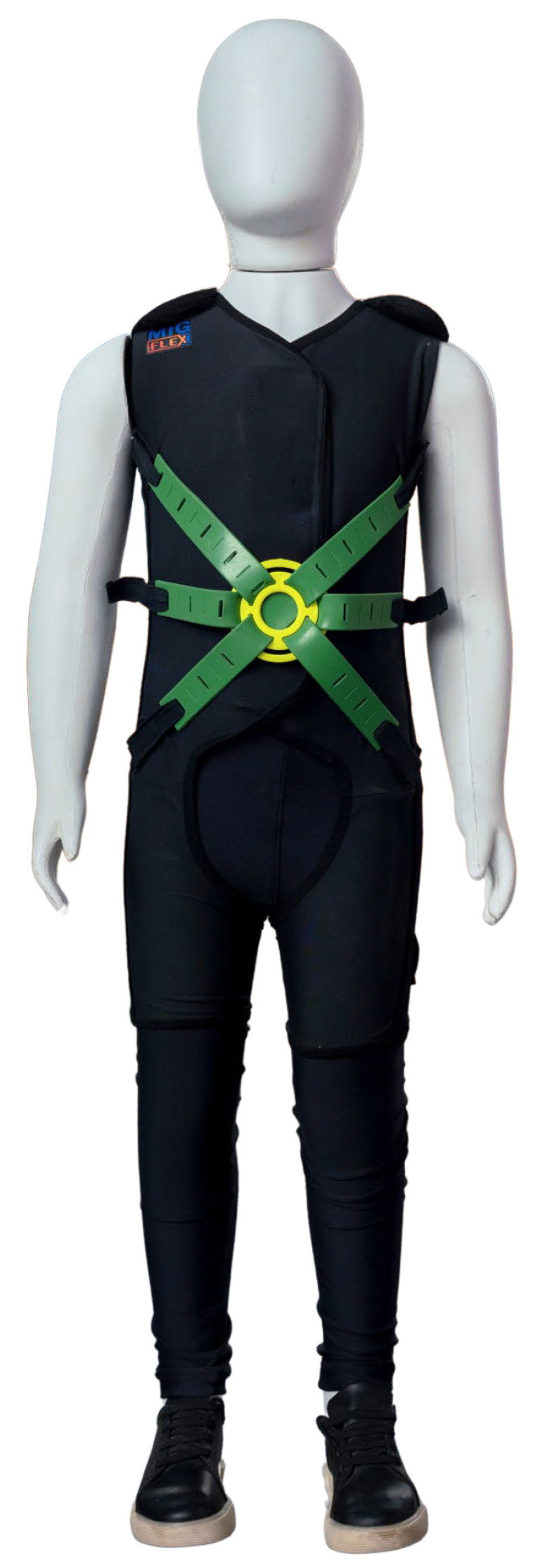
Therapeutic suit—MIG Flex.

MIG Flex is made up of a base suit, two anchors (also called “nodes”) and six viscoelastic straps (or myofascial straps). The base garment is made of polyamide (84%) and elastane (16%) and has adjustment systems with Velcro on the shoulders, chest, abdomen and thigh. In the navel region and lumbosacral region (L5–S1) there are anchors made of nylon that allow for the passage of the viscoelastic strip (represented by the green color in Figure 3). The viscoelastic strip is a non-toxic polymer (silicone) which has connection systems with the clips located on the side of the suit and which has paths similar to the myofascial lines (lateral line, spiral line and anterior and posterior functional lines). Accordingly, MIG Flex is a therapeutic suit made with flexible material which ensures that the child can carry out their activities without any mobility restrictions. MIG Flex is one of the components of the MIG program. Therefore, non-tolerance of the child’s use of clothing does not constitute a contraindication to intervention with the MIG program. The child can benefit from the other components of the program, such as skills training in the different units of the City of Tomorrow. The initial adjustment of MIG Flex to the child’s body is carried out gradually, according to tolerance. Furthermore, if the child demonstrates intolerance to the clothing, not only through oral language, but also through significant changes in behavior, adjustments to MIG Flex can be made gradually, until the child becomes accustomed to the clothing. Figure 3 illustrates MIG Flex.

In addition to the role of fascia in regulating movement, it is also important to highlight the role of interventions that engage both the superficial fascia (through the stimulation of their mechanical receptors) and the deep fascia (through the execution of movements) [223]. Active fascial training aims at elasticity and resilience, which provides effective muscle function with a greater degree of injury prevention [218]. An appropriate exercise load, applied regularly, can also favor a collagen architecture with a more “wavy” or crimped arrangement of fibers [226], resulting in a significant increase in elastic energy storage capacity [227]. Failure to exercise causes a loss of fiber elasticity and reduced slippage between them, forming tissue adhesions and even tangled, poorly organized arrangements [218,226]. This also impacts the quality of proprioceptive information sent to the central nervous system.

Fascial training, through movements in multiple ranges of extension and elasticity, aims to stimulate fibroblasts to maintain the architecture of collagen fibers with the potential for elastic storage [218]. Furthermore, as the fascia is rich in proprioceptive receptors, in the absence of tissue adhesions, the deep fascia is sensitive to both small joint movements and multi-joint movements [218]. It is believed that fascial sensitivity is greater than joint capsule sensitivity, as the receptors in the joint capsules are generally stimulated only in the extreme joint ranges and not during physiological movements, as occurs with fascial proprioceptive receptors [218,219,220].

Thus, in addition to postural stabilization, activities carried out with MIG Flex in a naturalistic space (City of Tomorrow) optimize functional postures as well as active and exploratory movement in the environment with enhanced muscle strength. The stability, postural compensation and active movement with enhanced muscle strength provided by the MIG Flex are provided by distributing stimulation along the functional, superficial, lateral, spiral and deep myofascial lines. The stimulation of these lines, carried out in a naturalistic environment, plays both mechanical and perceptual roles [219,220,221], thus contributing to the motricity and naturalistic environmental exploration carried out in the units that make up the City of Tomorrow. In the long term, the benefits of using the MIG Flex include sending correct proprioceptive information to the central nervous system and favoring the adjustment of muscle tone. Adjusting tone and promoting constant motor control leads to the patient executing correct movements. This correct execution of active movements provides greater quality to the fascial tissue, thus generating a positive cycle in the patient’s motor function.

To estimate the amount of time for the effects of using MIG Flex to result in permanent structural changes in the connective tissue, and, consequently, in the improvement of tissue quality, the period of tissue remodeling of collagen fibers must be considered. Connective tissue adapts to regularly imposed physiological tensions, whereas fibroblasts adjust their tissue matrices by adapting activity to favor demand. Thus, upon tension, fibroblasts adapt the arrangement of the collagen fiber network [228]. It is estimated that half of the collagen fibrils are replaced annually [229]. Extrapolating from these data, in six months, there will be a renewal of 30% of the collagen fibers, and in two years, there will be a renewal of 75% [218]. Therefore, an MIG Flex usage duration equivalent to three years is considered ideal. During this period, the renewal of collagen fibers would be completed, with tissue being adapted and effective in sending accurate proprioceptive information.

## 8. Intervention Results Assessment Strategy

The MIG approach is currently serving 206 children aged 1.8 years to 18.25 years with autism and their families in interdisciplinary clinics across Brazil. The support levels required by the children are: Level 1: 23.2%; Level 2: 37.4%; and Level 3: 30.5%. A substantial proportion of children present autism associated with genetic syndromes and other neurological conditions, such as cerebral palsy. Care is funded by private health insurance companies in accordance with Brazilian legislation [230]. Similar programs are beginning to be implemented by public health services, albeit in an incipient manner. Children receive physical, occupational and speech therapy in the same institution. Therapeutic goals are defined, and their implementation is collaboratively assessed with the families using the Canadian Occupational Performance Measure (COPM) [231]. The MIG program lasts for at least three years. Children receive 20 weekly hours of care, and parents participate in one weekly session of parent training. Psychological care is available for children and parents as needed. Some families experience such an overwhelming burden that they are unable to participate in the parent training program. In these cases, parents receive other forms of psychotherapy, usually individual. Children use the MIG Flex for 20 h of the week, as they are participating at the clinic. A new model of using the MIG Flex at home coupled with parent training is currently being investigated. The MIG Flex is well-accepted by most children, resulting in improvements in balance, gait, stereotypies and aggressive behaviors. 

In the current manuscript, we present the theoretical and methodological foundations of MIG. The next research step is to empirically assess the program’s validity. Table 3 describes the instruments that are being used for the initial assessment as well as for the evaluation of the results. The instruments listed in Table 3 are the main outcome measures. Additional instruments may be necessary, as appropriate in each case.

The validity of the program is currently being assessed through a series of single-subject, quasi-experimental studies using the ABAB design with triplets of participants. The next step is a randomized controlled trial.

## 9. Strengths and Limitations

The main strength of the present article is the addition of interventions, specifically focusing on motor manifestations in autism, from a predictive coding and embodied cognition perspective. At the same time that it adds specific interventions for motor manifestations, MIG preserves components that have been proven effective in other approaches, especially in naturalistic–developmental–behavioral ones. One limitation of this article is that it is restricted to presenting the theoretical–methodological framework of a new approach. Examination of the validity of this approach is ongoing. Another limitation is that the process of creating MIG was not based on a systematic review of the literature, but rather on the clinical and research expertise accumulated by authors with over 20 years in the rehabilitation of children with neurodevelopmental disorders and neurodevelopment, as well as 40 years in neuropsychological assessment and counseling, respectively.

## 10. Final Considerations

MIG was created in response to the different needs of people with autism. It consists of an integrated and interdisciplinary program of intervention strategies, considering the greatest possible number of levels of disability and functionality proposed by the WHO biopsychosocial model, as well as their interactions. MIG’s theoretical foundation is based on the analysis of behavior, cognitive neuropsychology and motor function, as well as motor cognition guiding the practice of professionals from different areas of healthcare and education. MIG’s entire theoretical foundation has resulted in the development of exclusive ingredients such as the City of Tomorrow and therapeutic suit MIG Flex. These ingredients favor environmental exploration by people with autism in a structured, naturalistic, active way, and are mediated by the MIG Flex. The support offered by therapeutic suit MIG Flex increases the ability to focus attention and understand what is happening in the environment, especially the social environment, favoring both motor and cognitive aspects related to social skills.

## Figures and Tables

**Table 1:** Characteristics of the Global Integration Method (MIG—Método de integração Global).

Component	Description	References
Evidence-based interdisciplinary practice	MIG is implemented in close collaboration among professionals from multiple specialties, making joint decisions with the family, and formulating a common intervention plan with measurable objectives and periodic monitoring of the achievement and reformulation of goals.	Atun-Einy et al., 2013 [57]; Bowman et al., 2021 [105]; Hume et al., 2021 [2]; Lustrea, 2019 [106]; Steinbrenner et al., 2020 [2].
Biopsychosocial approach	MIG uses interventions at the WHO’s five levels of biopsychosocial impacts.	Bolte et al., 2019 [43]; Schiariti et al., 2018 [44]; Silva et al., 2013 [45]; WHO, 2001 [46].
Family-centered practice and social validity	MIG implements a decision-making process in close collaboration with the family.	Almasri, An and Palisano, 2018 [127]; An et al., 2017 [128]; An et al., 2019. [129].
Bottom-up therapeutic approaches	MIG uses a flexible therapeutic suit based on myofascial trails with the aim of stabilizing postural tone, thus freeing up processing resources for socio-cognitive learning.	Borji et al., 2023 [130]; Jabouille et al., 2023 [131]; Leone et al., 2017 [132]; Reilly et al., 2008 [133]; Simmons et al., 2019 [134]; Torres and Denisova, 2016 [135]; Van Biesen et al., 2018 [136]; Wolpert et al., 2003 [137].
Top-down therapeutic approaches	MIG uses explicit instruction and development of cognitive schemas, imagery and sociomotor synchronization to promote social, emotional and cognitive skills, providing structure for action and decision making.	Ahar and Ghadiri, 2021 [138]; Kalyuga, 2010 [139]; Sweller et al.; 2011 [140].
Intensive and prolonged training	MIG assumes that significant results at the body structure and function level, involving tissue remodeling and activity-dependent neuroplasticity, require intense and prolonged intervention.	Bleyenheuft, et al., 2015 [141]; Figueiredo et al., 2020 [142]; Hodgson et al., 2022 [143]; Jackman et al., 2021 [144]; Sampaio et al., 2021 [145].
Naturalistic learning environment	MIG assumes that interventions in the child’s living environments or similar environments promote social learning and generalization.	Bruinsma et al., 2019 [8].
Concrete and playful materials and activities	MIG assumes that concrete and playful materials and activities promote motivation and engagement in interventions.	Bruinsma et al., 2019 [8].
Parent-mediated interventions	MIG assumes that parent-mediated interventions facilitate generalization in the child’s living environment and reduce costs.	Ingersoll and Dvortcsak, 2019 [146].
Manualization	MIG operationalizes its procedures through teaching materials provided to professionals.	Odom et al., 2010 [122]
Supervision and continued education	MIG offers online supervision to professionals and continues training through regularly held courses and conferences.	Odom et al., 2010 [122]
Care management through app	MIG uses an mobile app (MIG+) especially developed to manage interdisciplinary healthcare efficiently. MIG is based on the assumption that the agility and integration of information transmitted by the family and the technical team positively impact healthcare.	Fenning et al., 2022 [147].

**Table 2:** City of Tomorrow’s naturalistic learning units.

Purpose	Unit	Description	Theoretical–Methodological Foundation	References
Logistics	Therapeutic suit (MIG Flex) mounting space	Spaces to store and assemble the flexible therapeutic suit (MIG Flex).	Promoting postural stability, adequate regulation of muscle tone and quality of movements performed.	Loffi, 2019 [149]; Stecco et al., 2023 [150].
Environmental reactivity workup	Stimulus–control, controlled instability, and fixation rooms	Spaces to work on reducing distractors and focus and attention activities to achieve partial independence.	Graded exposure and desensitization for hypersensitivity and attention training	Hadad and Schwartz, 2019 [151]; Williams et al., 2021 [152].
Infant intervention unit	Baby room	Early intervention	Early intervention	Damiano and Forssberg, 2021 [153]; Grzadzinski et al., 2018 [69].
Naturalistic learning units	Home	Daily living activities and autobiography	The child works on daily life skills (such as eating, dressing, personal hygiene, using the bathroom, brushing teeth and taking a shower, for example) and, at the same time, prepares their autobiography. Daily living skills training is based on the cognitive–behavioral model of self-instruction in problem solving. Autobiographical elaboration allows the child to incorporate their disabilities as a part of their self and, at the same time, develop a positive self-concept.	Fivush, 2011 [154]; Nelson and Fivush, 2004 [155]; Kendal and Braswell, 1993 [156]; Nezu and Nezu, 2020 [157]; Santacreu, 2020 [158].
	Supermarket	Shopping at the supermarket	The cognitive supermarket shopping script allows the child develops linguistic and conceptual skills of categorization and vocabulary, numerical–arithmetic, social and planning skills.	Crick and Dodge, 1994 [159]; Dodge, 1993 [160]; Fritz et al., 2013 [161]; Freitas et al., 2022 [162]; Kendal and Braswell, 1993 [156]; Nezu and Nezu, 2020 [157]; Santacreu, 2020 [158]; Ziv et al., 2014 [163].
	School, Castle of Letters and Numbers	Oral lexical comprehension and expression Reading and writing words and numbers	Oral and written activities related to words and numbers are based on cognitive–neuropsychological models of lexical processing.	Ellis et al., 1994 [164]; Friedmann and Coltheart, 2018 [165]; Moura et al., 2021 [166]; Temple, 1997 [167].
	Castle of Tales	Oral narratives	The child learns to analyze and elaborate upon oral narratives based on the categories of story grammar. Oral narratives constitute an important precursor to reading comprehension.	Dawes et al., 2019 [168]; Hessling and Schuele, 2020 [169]; Saywitz and Snyder, 1996 [170]; Spencer et al., 2013 [171].
	Desenhix (“Drawix”)	Visuospatial, visuoconstructive and graphomotor skills underlying drawing. Figurative representation of the human body. Graphic narratives.	The child learns to use graphic strategies to represent the human body, emotions and social situations created in the form of comics.	Fivush, 2011 [154]; Grossi and Trojano, 1999 [172]; Roncato et al., 1987 [173]; Nelson & Fivush, 2004 [155]; Saywitz and Snyder, 1996 [170]; Van Sommers, 1984 [174]; Van Sommers, 1989, [175].
	Fitness space (sports court) and Social Rules Gym	Basic sports skills and participation in team sports and rule-based social/cooperative games.	Operant conditioning and modeling strategies are used to develop basic sports skills and participation in group sports.	Wang et al., 2022 [4]; Vincent et al., 2018 [176]; Ketcheson et al., 2017 [177].
	Galaxy of the Future	Role-playing game.	Participation skills, group strategies and development of gesture imitation skills are developed through role-playing games.	Ke and Moon, 2018 [178]; Prince, 2018 [179]; Tsai et al., 2021 [180].

**Table 3:** Assessment instruments.

Domain	Instrument	Description	Reference
Autism symptoms	Social Responsiveness Scale (SRS-2)	The SRS-2 aims to assess symptoms related to autism spectrum disorder (ASD) as well as to classify them into mild, moderate and severe levels. Its assessment is made globally and specifically through six subcategories of symptoms. These subcategories are social perception, social cognition, social communication, social motivation, restrictive and repetitive patterns and social communication and interaction.	Constantino and Gruber (2012) [232]; Borges and Hauck-Filho (2020) [233]
Support level	Autism Classification System of Functioning: Social Communication (ACSF:SC)	ACSF is a five-level descriptive system based on the International Classification of Functioning, Disability and Health (ICF) that provides a standardized way to report a child’s/youth’s social communication abilities in two situations: when they are performing at their best (capacity) and how they usually perform (typical performance).	Di Rezze et al., 2016 [234]
General cognitive ability	Raven’s CPM	CPM is a 36-item test used to estimate the nonverbal reasoning of children aged from 6 years to 11 years and 11 months.	Angelini et al., 1999 [235]; Raven, Raven and Court, 2018 [236]
Behavioral disorders	Strengths and Difficulties Questionnaire (SDQ)	The SDQ is a brief behavioral screening questionnaire for 2–17-year-olds, and is divided into five subscales: prosocial behavior problems, hyperactivity, emotional, behavioral and relationship problems.	Goodman, 1997 [237]; Fleitlich and cols., 2000 [238]
Family needs and priorities	Canadian Occupational Performance Measure (COPM)	The COPM is a client-centered outcome measure to identify and prioritize everyday issues that restrict the participation of individuals. This measure focuses on self-care, leisure and productivity.	Law et al., 2005 [231]
Activity and participation	Pediatric Evaluation of Disability Inventory Computer Adaptive Test (PEDI-CAT)	The PEDI-CAT is a computer adaptive caregiver report which measures daily activities, mobility, social/cognitive and responsibility with the aim of identifying functional delay and examining the improvement of an individual child after intervention.	Haley et al., 2012 [239]
Motor abilities	Pediatric balance scale (PBS)	PBS is a 14-item, criterion-referenced measure based on the Berg Balance Scale that examines functional balance in the context of everyday tasks in the pediatric population.	Franjoine et al., 2003 [240]
	Inertial sensor of standing balance (linear and angular oscillations, BaiobitTM)	This is a tool used for measuring postural oscillations of the center of mass (CM) during bipedal balance or single-pedal position, eyes open or closed, to evaluate the patient’s postural control. The main variables measured using this unit are: (a) the area/surface of the ellipse, (b) path length, (c) displacement amplitude and (d) speed.	Paillard and Noé, 2015 [241]
	Timed Up and Go (TUG)	A tool used in clinical practice with children and teenagers to assess gait and dynamic balance.	Martín-Díaz et al., 2023 [242]
	Test of gross motor development (TGMD-2)	The TGMD-2 is a discriminative, norm-referenced test used to assess the level of competence in the motor skills involving large muscle groups that produce the force needed to move the trunk and upper and lower limbs.	Ulrich, Soppelsa and Albaret, 2000 [243]
	Nine-hole peg test (9HPT)	9HPT is a standardized, quantitative assessment used to measure finger dexterity.	Poole et al., 2005 [244]

## Data Availability

No new data were created or analyzed in this study. Data sharing is not applicable to this article.

## Supplementary Materials

The following supporting information can be downloaded at: https://www.mdpi.com/article/10.3390/children11020191/s1.
