# Supplementary material for: Theoretical–Methodological Foundations for the Global Integration Method (Método de Integração Global—MIG) in the Treatment of Autism Spectrum Disorder
Source: Children (Basel). 2024 Feb 2;11(2):191. doi: 10.3390/children11020191 (PMC10887636; doi:10.3390/children11020191)
Supplement: Supplementary file 1 [file children-11-00191-s001.zip › children-2802182-supplementary.pdf]

Supplementary Material 1

Terms	Concept
Sensory hyporeactivity	Failure or inability to react to stimuli and the search for or the self-stimulation of certain sensory experiences, generally referred to as sensory seeking behaviors.
sensory hyperreactivity	Exacerbated reaction to stimuli.
Predictive coding	The brain uses the senses to check the continuing usefulness and survival value of its own predictions about the world.
Embodied cognition	The concept suggests that many features of cognition are shaped by motor and sensory processes.
Cognitive schemas	Patterns of thought and behavior used by the brain to organize information about the world. They are built from our memories of unique experiences and stored in long-term memory.
Cognitive load theory	It is an instructional theory that explains how the brain processes and stores information.It suggests that our working memory is only able to hold a small amount of information at any one time.
Motor ressonance	The ability to directly understand (i.e., motor-based understanding) and synchronize with others' behavior.
Motor interference	A more general and pervasive motor marker of social anomalies (i.e.,the observation of a movement may interfere with the simultaneous execution of a congruent or incongruent movement).
Myofascial trains	Also called "anatomy trains", myofascial trains are a set "mapping" of whole body fascia and myofascial linkages (throughout the whole body, from the head to the toes) subserving complex movement synergies.
Tensegrity	A tensegrity system consists of an intrinsically stable system that contains a group of components in compression, within a network of interconnected components under continuous tension
